# Synuclein Deficiency Results in Age-Related Respiratory and Cardiovascular Dysfunctions in Mice

**DOI:** 10.3390/brainsci10090583

**Published:** 2020-08-24

**Authors:** Patrick S. Hosford, Natalia Ninkina, Vladimir L. Buchman, Jeffrey C. Smith, Nephtali Marina, Shahriar SheikhBahaei

**Affiliations:** 1 Department of Neuroscience Physiology and Pharmacology, Center for Cardiovascular and Metabolic Neuroscience, University College London (UCL), London WC1E 6BT, UK; p.hosford@ucl.ac.uk (P.S.H.); n.marina@ucl.ac.uk (N.M.); 2 School of Biosciences, Cardiff University, Cardiff CF10 3AX, UK; ninkinan@cf.ac.uk (N.N.); buchmanvl@cf.ac.uk (V.L.B.); 3 Institute of Physiologically Active Compounds, Russian Academy of Sciences (IPAC RAS), 1 Severniy proezd, 142432 Chernogolovka, Moscow Region, Russia; 4 Cellular and Systems Neurobiology Section, National Institute of Neurological Disorders and Stroke (NINDS), National Institutes of Health (NIH), Bethesda, MD 20892, USA; SmithJ2@ninds.nih.gov; 5 Neuron-Glia Signaling and Circuits Unit, National Institute of Neurological Disorders and Stroke (NINDS), National Institutes of Health (NIH), Bethesda, MD 20892, USA

**Keywords:** aged mouse, baroreflex, hypoxia, hypercapnia, Parkinson’s disease, synuclein

## Abstract

Synuclein (α, β, and γ) proteins are highly expressed in presynaptic terminals, and significant data exist supporting their role in regulating neurotransmitter release. Targeting the gene encoding α-synuclein is the basis of many animal models of Parkinson’s disease (PD). However, the physiological role of this family of proteins in not well understood and could be especially relevant as interfering with accumulation of α-synuclein level has therapeutic potential in limiting PD progression. The long-term effects of their removal are unknown and given the complex pathophysiology of PD, could exacerbate other clinical features of the disease, for example dysautonomia. In the present study, we sought to characterize the autonomic phenotypes of mice lacking all synucleins (α, β, and γ; αβγ^−/−^) in order to better understand the role of synuclein-family proteins in autonomic function. We probed respiratory and cardiovascular reflexes in conscious and anesthetized, young (4 months) and aged (18–20 months) αβγ^−/−^ male mice. Aged mice displayed impaired respiratory responses to both hypoxia and hypercapnia when breathing activities were recorded in conscious animals using whole-body plethysmography. These animals were also found to be hypertensive from conscious blood pressure recordings, to have reduced pressor baroreflex gain under anesthesia, and showed reduced termination of both pressor and depressor reflexes. The present data demonstrate the importance of synuclein in the normal function of respiratory and cardiovascular reflexes during aging.

## 1. Introduction

Synucleins (α, β, and γ) are a family of highly-conserved vertebrate-specific proteins, specifically enriched in pre-synaptic terminals of neurons and thought to be involved in regulating vesicular release and recycling of neurotransmitters [1,2,3]. The alpha-synuclein (α-syn) member of this family has come under intense research scrutiny because the pathophysiology of Parkinson’s disease (PD) is particularly linked with α-syn abnormalities due to the discovery of a rare form of familial PD that was mapped to a single nucleotide change in the α-syn gene [4]. Subsequently, genome-wide association studies (GWAS) found α-syn to be the most relevant gene in cases of idiopathic PD. This led to the development of numerous animal models of PD based on the mutation or over-expression of α-syn [5]. Additionally, there is an important role of α-syn in regulation of key stages of dopamine release, reuptake and recycling [6]. However, despite playing such an important role in pathology, surprisingly little was known about the physiological role of this protein. This was, in part, due to the sequence homology of the genes encoding the three members of this protein family [7,8]. The pattern of expression, predominantly in neuronal tissues, of these three proteins also has significant overlap, and while some tissues may express more than one member, generally all three are expressed simultaneously [9]. Synucleins seem to operate in redundancy as in single or double syn-knockout mice display decline in striatal dopamine and impaired synaptic function over time [10,11,12]. However, αβγ-syn triple-knockout (αβγ^−/−^) mice displayed a more severe age-dependent phenotype in comparison to single or double knockouts [2,7,13,14].

Autonomic deficits in PD are well-known to contribute to the overall pathology [15,16,17] but have recently received renewed attention because this dysfunction may precede the onset of motor symptoms [18,19,20]. Understanding how autonomic dysfunction associated with PD can be better treated or used for diagnosis requires studies with animal models. Indeed, both toxin-based animal models and those that rely on the (over)expression of (human) α-syn have been shown to recapitulate many of the features of PD dysautonomia [21]. Recently, reducing α-synuclein level has gained attention as a therapeutic method to limit the progression of PD [12]; however, experimental evidence on the long-term effects of removing α-synuclein on the development and normal function of the autonomic nervous system is lacking. Accordingly, in the present study, we sought to characterize the respiratory and cardiovascular phenotype of αβγ^−/−^ animals in order to better understand the role of this protein family in homeostatic autonomic cardiorespiratory responses.

## 2. Methods

### 2.1. Ethical Approval

All experiments were performed in accordance with the European Commission Directive 86/609/EEC (European Convention for the Protection of Vertebrate Animals used for Experimental and Other Scientific Purposes), the UK Home Office (Scientific Procedures) Act (1986), and the US National Institutes of Health Guide for the Care and Use of Laboratory Animals, with project approval from the respective Institutional Animal Care and Use Committees.

### 2.2. Generation of Single-, Double- and Triple-Synuclein-Null Mutant Animals

Generation of the αβγ-synuclein triple-knockout (αβγ^−/−^) mouse line has been described previously [14]. All animals were on the same C57Bl/6 genetic background. Briefly, lines of single synuclein knockout animals were backcrossed with C57Bl/6J mice for more than eight generations. Intercrossing the resultant animals produced double synuclein knockout and finally, αβγ^−/−^ mice. Wild-type (WT) littermates were used as controls. Young (3–4 months) and aged (18–20 months) male mice used in this study were housed in a temperature-controlled facility with a normal light–dark cycle (12 h:12 h, lights on at 7:00 am). Access to food (standard laboratory rodent diet) and water was provided ad libitum.

### 2.3. Physiological Experiments in Conscious Mice

#### 2.3.1. Non-Invasive Breathing Measurement

Respiratory parameters were measured in unrestrained conscious mice by whole-body plethysmography as described previously [22,23]. Briefly, the mouse was placed in a Plexiglas recording chamber (~200 mL) that was flushed continuously with room air (unless otherwise required by the protocol) at a rate of 0.3 L min^−1^. The animals were allowed to acclimatize to the chamber environment for ~30 min in room air. After baseline ventilation was measured, the O_2_ concentration in the chamber and thus inspired air was reduced to 15% and 10% for 5 min at each O_2_ level. Similarly, in separate experiments, hyperoxic (~33% O_2_) hypercapnia was induced by incrementally increasing the concentration of CO_2_ in the chamber respiratory gas mixture to 3%, 6%, and 9%. Each CO_2_ concentration was maintained for 5 min. Concentrations of CO_2_ in the chamber were monitored online using a fast-response O_2_/CO_2_ analyzer (Morgan Medical, Hertford, UK). Respiratory rate (ƒ_R_, breaths per minute) and tidal volume (*V*_T_*,* normalized per kilogram of body weight) were determined by the pressure changes in the chamber as described before [23,24]. The pressure signal was amplified, filtered, recorded, and analyzed offline using *Spike2* software (Cambridge Electronic Design, Cambridge, UK). The measurements of these ventilatory variables were obtained during the last 1–2 min of baseline recordings (i.e., before exposure to the gas mixtures) and during a 2 min period near the termination of each hypoxia or hypercapnia stimulus when the breathing pattern was stabilized. Hypoxia- or hypercapnia-induced changes in ƒ_R_, *V*_T_, and minute ventilation (*V*_E_ *=* ƒ_R_ × *V*_T_) were analyzed from the recordings.

#### 2.3.2. Non-Invasive Blood Pressure Measurement

Non-invasive blood pressure measurements were conducted in conscious mice of all strains by means of an integrated tail-cuff plethysmography system (CODA system, Kent Scientific, Torrington, CT, USA; [25]). Blood pressure measurements were performed by the same investigator and at the same time of day for all mice within each experimental group. Mice were moved to a quiet, temperature-controlled area 12 h before measurements commenced. Air temperature was between 32 and 34 °C, thermoneutral for rodents, in order to ameliorate increases in sympathetic drive associated with cold stress [26]. Animals were restrained in a tube with nose-cone attachment (Kent Scientific) and placed on a heated platform at 37 °C. The tail was instrumented with a cuff and volume-pressure recording sensor. At least ten consecutive volume–pressure measurements were taken over a period of 15 min and averaged to determine resting arterial blood pressure and heart rate. Measurements were repeated the following day and all variables reported are an average of two consecutive measurement periods.

### 2.4. Anesthetized Preparation for Baroreflex Measurements

#### 2.4.1. Surgical Preparation

Mice were anesthetized with urethane (ethyl carbamate; 1.2 g kg^−1^, i.p.) and both the left common carotid artery and right jugular vein were cannulated using fine-bore polyethylene tubing (ID.38 OD 1.09 mm; Smith’s Medical, Kent, UK) filled with heparinized saline. Mice were allowed to freely breath room air and placed on a homeothermic heating pad to maintain body temperature at 37 ± 5 °C. The arterial cannula was connected to a pressure transducer for measurement of blood pressure. Depth of anesthesia was assessed by absence of withdrawal from paw-pinch and the stability of blood pressure and heart rate recorded. Additionally, two subcutaneous electrodes were placed on left and right flanks for recording of ECG. Signals were amplified and filtered (High-pass 50 Hz, low-pass 20 kHz) using a Neurolog System (Digitimer, Wellyn Garden City, UK). Recordings of the physiological variables obtained in anesthetized animals were digitized using a Cambridge Electronic Design (CED) power1401 interface and analyzed using *Spike2* software.

#### 2.4.2. Baroreflex Protocol

After allowing the animal to stabilize approximately 10 min following surgery, the baroreflex protocol was started. Animals were subjected to three randomized doses of phenylephrine (5, 10 and 25 μg per animal) and/or sodium nitroprusside (5, 15 and 30 μg per animal). Drugs were administered slowly over 5–10 s via the jugular vein cannula in volumes that did not exceed 50 μL. Maximum changes in mean arterial blood pressure and R-R interval were calculated from the blood pressure and ECG trace, respectively. Baroreflex gain was calculated by the change in heart rate divided by the change in blood pressure. Additionally, the ‘overshoot’ in heart rate and systolic blood pressure after returning to baseline following activation of the pressor reflex and depressor reflexes was assessed.

#### 2.4.3. Morphometries

At the end of the experiment, animals were humanely sacrificed with an overdose of pentobarbital sodium (30 mg kg^−1^). The heart was rapidly removed and rinsed with saline. Total heart weight was measured and then the left ventricle was carefully dissected free of the remaining tissue under microscopic guidance and weighed.

### 2.5. Data Analysis

Data are reported as mean ± SEM and plotted as individual points using Prism 8 (GraphPad Inc., San Diego, CA, USA). Datasets were tested for normality using a Shapiro–Wilk normality test and were compared by Kruskal–Wallis ANOVA by ranks, Mann–Whitney *U* rank test, or Wilcoxon matched-pairs signed-rank test as appropriate. Differences between groups with *p* < 0.05 were considered significant.

## 3. Results

### 3.1. Respiratory Activity in Conscious Mice

#### 3.1.1. Resting Breathing in Room Air and Hyperoxic Condition

**Figure 1:**
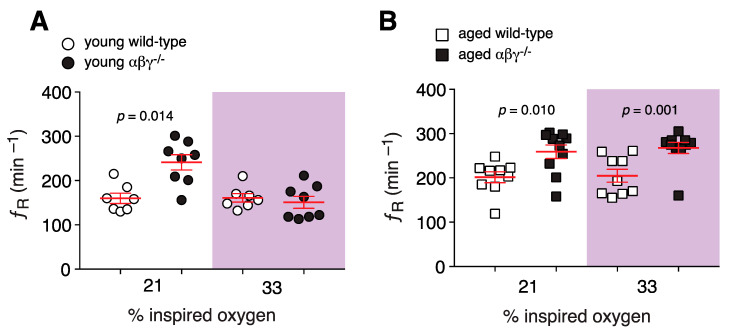
αβγ^−/−^ mice display age-dependent increases in resting breathing frequency in normoxia and hyperoxia. (**A**,**B**) Population data from whole-body plethysmography measurements showing respiratory rate (ƒ_R_) in young (**A**) and aged (**B**) WT and αβγ^−/−^ mice at room air (21% O_2_) and hyperoxic conditions (33% O_2_). *p*—Mann–Whitney *U* rank test.

The resting breathing frequency (ƒ_R_) of young αβγ^−/−^ mice in room air (i.e., normoxia/normocapnia) was higher when compared to the wild-type mice (241 ± 17 min^−1^ (*n* = 8) vs. 166 ± 11 min^−1^ (*n* = 7); *p* = 0.014, Mann–Whitney *U* rank test; Figure 1A). Similarly, with αβγ^−/−^ aged mice, ƒ_R_ remained higher (259 ± 15 min^−1^ (*n* = 10) vs. 195 ± 13 min^−1^ (*n* = 9) than age-matched wild-type mice; *p* = 0.010; Figure 1B). However, in the hyperoxia/normocapnia condition (~33% O_2_ in the inspired air), ƒ_R_ was not different in younger mice (245 ± 6 min^−1^ (*n* = 8) vs. 251 ± 8 min^−1^ (*n* = 7); *p* = 0.44, Mann–Whitney *U* rank test; Figure 1A), but in this condition, ƒ_R_ remained higher (by 24%) in aged αβγ^−/−^ mice (268 ± 13 min^−1^ (*n* = 10) vs. 204 ± 15 min^−1^ (*n* = 9); *p* = 0.001, Mann–Whitney *U* rank test; Figure 1B).

#### 3.1.2. Ventilatory Response to Hypoxia

**Figure 2:**
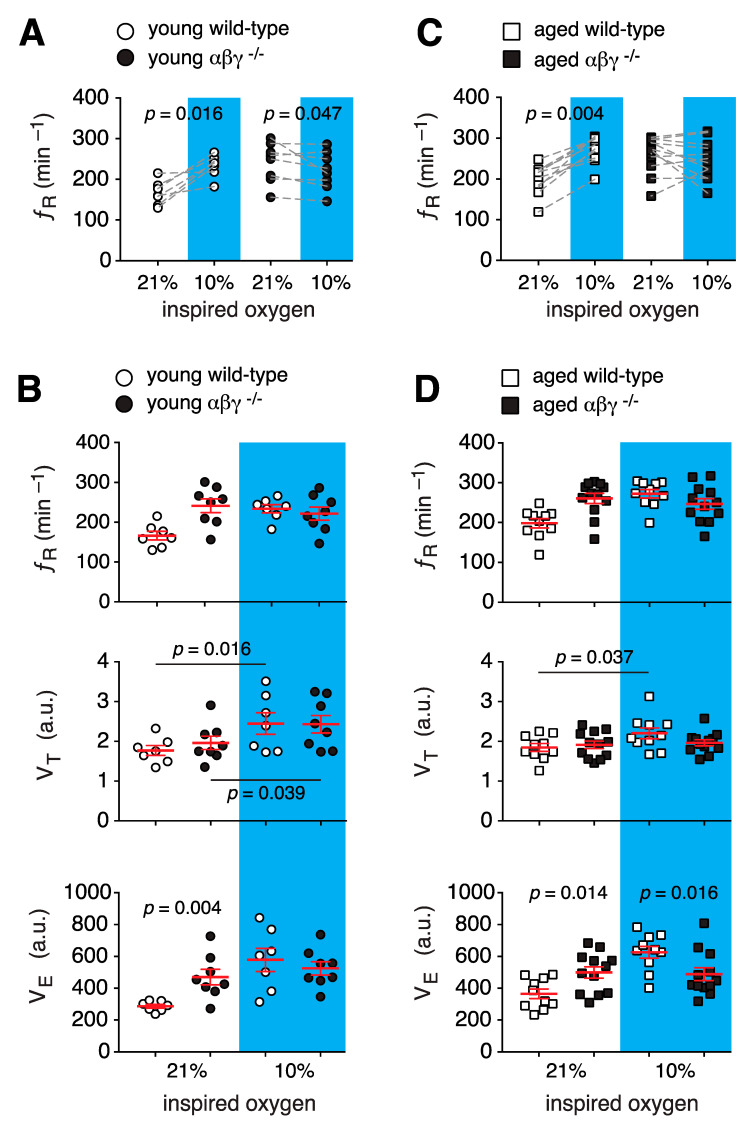
Respiratory responses of αβγ^−/−^ mice to hypoxia. (**A**,**B**) Group data from whole-body plethysmography measurements of respiratory rate (ƒ_R_), tidal volume (*V*_T_), and minute ventilation (*V*_E_) of unrestrained young wild-type and αβγ^−/−^ mice in room air (21% O_2_) and moderate hypoxia (10% O_2_). (**C**,**D**) Summary data from whole-body plethysmography measurements of breathing parameters (ƒ_R_, *V*_T_, *V*_E_) of aged wild-type and αβγ^−/−^ mice in 21% and 10% O_2_. *p*—Wilcoxon matched-pairs signed-rank test (**A**,**C**), middle panels in (**B**,**D**); Mann–Whitney *U* rank test (lower panels in (**B**,**D**)).

ƒ_R_, *V*_T_, and *V*_E_ responses during mild hypoxic challenges (15% O_2_ in the inspired air) were not different in young or aged transgenic mice when compared to their wild-type counterparts (data not shown). However, when challenged with moderate hypoxia (10% O_2_ in the inspired air), ƒ_R_ increased in young wild-type mice by ~41% (234 ± 10 min^−1^ vs. 166 ± 11 min^−1^ in room air, *n* = 7; *p* = 0.016, Wilcoxon matched-pairs signed-rank test; Figure 2A). In contrast, during hypoxia, ƒ_R_ decreased in young αβγ^−/−^ animals (by ~8%, 222 ± 17 min^−1^ vs. 241 ± 16 min^−1^ in room air, *n* = 8; *p* = 0.047, Wilcoxon matched-pairs signed-rank test; Figure 2A). Despite this decrease in ƒ_R_, the hypoxia-induced ƒ_R_ in young αβγ^−/−^ animals was not different from wild-type (*p* = 0.7, Mann–Whitney *U* rank test; Figure 2B). Hypoxic challenge increased tidal volume (*V*_T_) by ~37% in wild-type (2.5 ± 0.2 a.u. vs. 1.8 ± 0.1 a.u. in normoxia; *p* = 0.016, Wilcoxon matched-pairs signed-rank test) and by ~20% in αβγ^−/−^ animals (2.4 ± 0.2 a.u. vs. 1.8 ± 0.1 a.u. at normoxia, *p* = 0.039, Wilcoxon matched-pairs signed-rank test; Figure 2B). However, the magnitude of the hypoxic *V*_T_ was not different between groups. In addition, hypoxia-induced minute ventilation (*V*_E_) was quantitatively similar between young wild-type and αβγ^−/−^ mice (525 ± 42 a.u. vs. 578 ± 73 a.u. in wild-type; *p* = 0.5, Mann–Whitney *U* rank test; Figure 2B).

In aged αβγ^−/−^ animals, hypoxia did not affect ƒ_R_ (259 ± 15 min^−1^ vs. 260 ± 12 min^−1^ in room air, *n* = 10; *p* = 0.77, Wilcoxon matched-pairs signed-rank; Figure 2C). Although hypoxia increased ƒ_R_ in aged wild-type mice by ~37% (271 ± 12 min^−1^ vs. 195 ± 13 min^−1^ in room air, *n* = 9; *p* = 0.004, Wilcoxon matched-pairs signed-rank; Figure 2C), the hypoxic breathing rate was not different between aged αβγ^−/−^ and wild-type mice (260 ± 12 min^−1^ vs. 271 ± 12 min^−1^ in wild-type; *p* = 0.55, Mann–Whitney *U* rank test; Figure 2D). During hypoxia *V*_T_ was also increased in aged wild-type mice by ~19% (2.2 ± 0.1 a.u. vs. 1.9 ± 0.1 a.u. at normoxia, *n* = 10; *p* = 0.037, Wilcoxon matched-pairs signed-rank test). However, hypoxia did not cause a significant increase in *V*_T_ in aged αβγ^−/−^ animals (1.97 ± 0.1 a.u. vs. 1.91 ± 0.1 a.u. at normoxia, *n* = 12; *p* = 0.52, Wilcoxon matched-pairs signed-rank test; Figure 2B). As opposed to younger mice, hypoxia-induced *V*_E_ was decreased in aged αβγ^−/−^ animals (513 ± 44 a.u. vs. 651 ± 31 a.u. in wild-type; *p* = 0.016, Mann–Whitney *U* rank test; Figure 2B).

#### 3.1.3. Ventilatory Response to Hypercapnia

**Figure 3:**
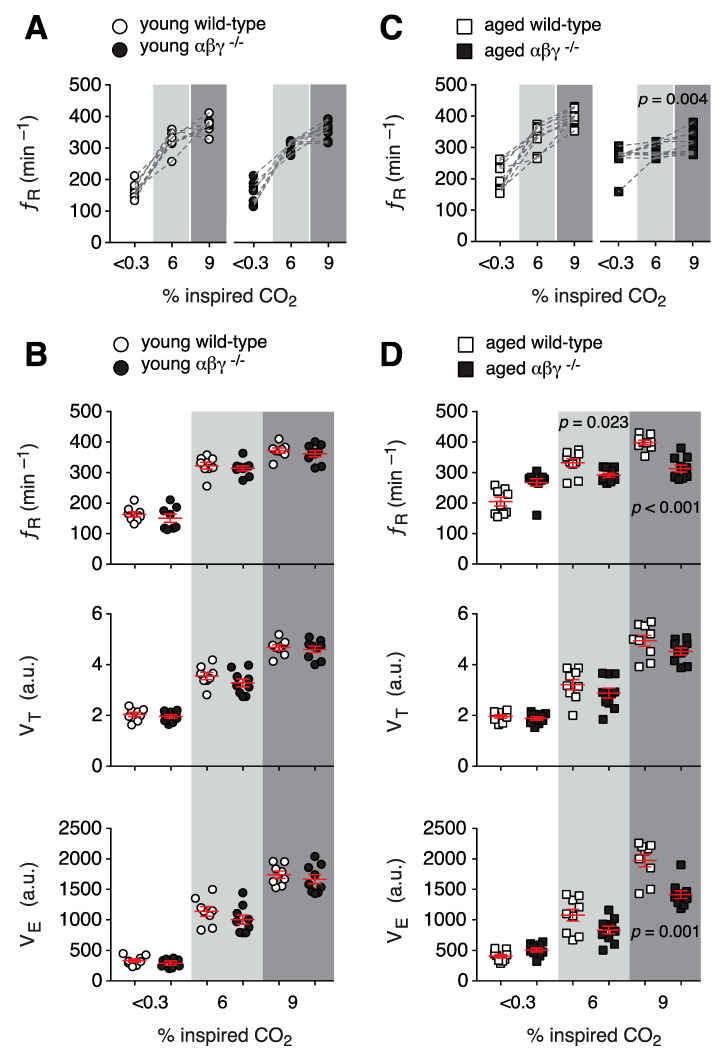
Respiratory responses of αβγ^−/−^ mice to hypercapnia. (**A**,**B**) Group data from whole-body plethysmography measurements of respiratory rate (ƒ_R_), tidal volume (*V*_T_), and minute ventilation (*V*_E_) of unrestrained young wild-type and αβγ^−/−^ mice in < 0.03% CO_2_, mild (6% CO_2_), and moderate hypercapnia (9% CO_2_). (**C**,**D**) Summary data from whole-body plethysmography measurements of breathing parameters (ƒ_R_, *V*_T_, *V*_E_) of aged wild-type and αβγ^−/−^ mice in < 0.03%, 6%, and 9% CO_2_. *p*—Wilcoxon matched-pairs signed-rank test (**C**); Mann–Whitney *U* rank test (**D**).

ƒ_R_, *V*_T_, and *V*_E_ responses during mild hypercapnic challenges (3% CO_2_ in the inspired air) were not different in young or aged transgenic mice when compared to their wild-type counterparts (data not shown). Similarly, there were no differences in respiratory responses to moderate hypercapnia challenge (6% CO_2_ in the inspired air) and severe hypercapnia challenge (9% CO_2_ in the inspired air) between young αβγ^−/−^ and wild-type mice (Figure 3A,B). However, compared to aged-wild-type mice, aged αβγ^−/−^ mice displayed impaired CO_2_-induced augmentation of ƒ_R_ in 6% CO_2_ (291 ± 7 min^−1^ vs. 268 ± 13 min^−1^ in normocapnia, *n* = 10; *p* = 0.08, Wilcoxon matched-pairs signed-rank test) and 9% CO_2_ (313 ± 11 min^−1^ vs. 268 ± 13 min^−1^ in normocapnia, *n* = 10; *p* = 0.004, Wilcoxon matched-pairs signed-rank test; Figure 3C). In addition, CO_2_-induced ƒ_R_ in aged αβγ^−/−^ mice was found to be lower by 12% [291 ± 7 min^−1^ (*n* = 10) vs. 332 ± 13 min^−1^ in wild-type (*n* = 9); *p* = 0.023, Mann–Whitney *U* rank test] and 20% [313 ± 11 min^−1^ (*n* = 10) vs. 398 ± 8 min^−1^ in wild-type (*n* = 9); *p* < 0.001, Mann–Whitney *U* rank test], respectively (Figure 3D).

In addition, the CO_2_-induced increase in *V*_E_ was lower in aged αβγ^−/−^ mice when challenged with 6 and 9% CO_2_ by 23% (834 ± 62 a.u. vs. 1077 ± 97 a.u. in wild-type; *p* = 0.07, Mann–Whitney *U* rank test) and 28% (1414 ± 63 a.u. vs. 1974 ± 105 a.u. in wild-type; *p* = 0.001, Mann–Whitney *U* rank test; Figure 3D), respectively.

### 3.2. Heart Morphometry Parameters

Heart morphometry measurements were taken from both aged and young αβγ^−/−^ mice. Body, heart and left ventricle weights were recorded and heart to body weight ratio was calculated. No significant differences (*p* > 0.05, Mann–Whitney *U* rank test) between phenotypes in either age groups were detected (Table 1).

### 3.3. Hemodynamics in Conscious Mice

In young animals, no differences in blood pressure or heart rate were detected between genotypes in any variable measured (*p* > 0.05, Mann–Whitney *U* rank test; Table 2). However, resting systolic, diastolic and MAP were all significantly (*p* = 0.015, Mann–Whitney *U* rank test) higher in aged αβγ^−/−^ compared to wild-type animals (Table 2), though basal heart rate was not different between genotypes in aged mice. Furthermore, we found no significant difference in hemodynamic measurements between single synuclein-null (α^−/−^, β^−/−^, γ^−/−^) and the double synuclein-null (αγ^−/−^) animals when compared to the aged control group (Table 3).

Interestingly, we found both aged wild-type and the TKO mice were significantly hypertensive compared to their young counterparts. Resting systolic, diastolic and MAP, but not heart rate, were all significantly (*p* < 0.001, Mann–Whitney *U* rank test) higher in the aged vs. the young wild-type mice.

### 3.4. Hemodynamics under Anesthesia

Systolic, diastolic, mean arterial pressure and heart rate were measured over a 5-min period immediately prior to commencement of the baroreflex protocol. Under anesthesia, there was no significant difference detected in any parameter between genotypes (*p* < 0.05; Mann–Whitney *U* rank test; Table 4).

### 3.5. Baroreflex

#### 3.5.1. Baroreflex Sensitivity

**Figure 4:**
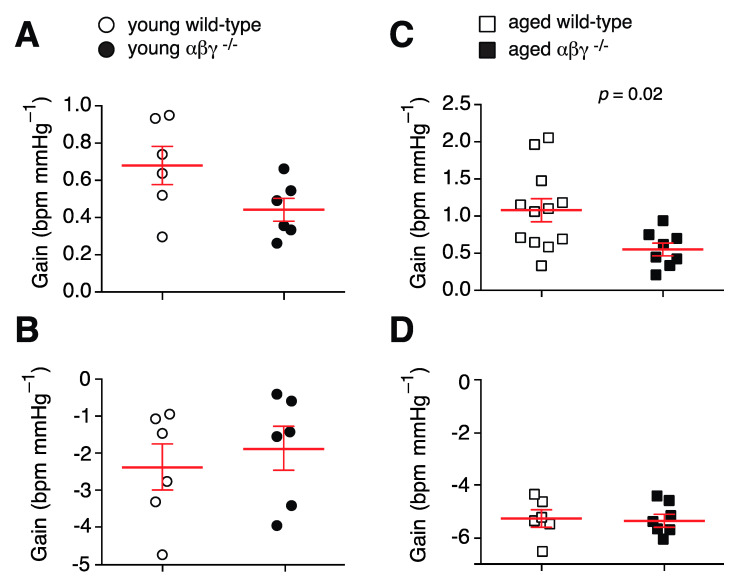
Comparisons of baroreflex gain in wild-type and αβγ^−/−^ mice. (**A**) Depressor reflex in aged mice. (**B**) Pressor reflex in aged mice. (**C**) Depressor reflex in young mice. (**D**) Pressor reflex in young mice. Means ± S.E.M were compared with Mann–Whitney *U* rank test.

Baroreflex sensitivity was assessed over a range of blood pressure changes from −30 to +50 mmHg (Figure 4). No significant differences in either the pressor or the depressor reflex were detected in the young animals between genotypes (Figure 4A,B). In aged animals, the pressor reflex was found to be significantly attenuated in αβγ^−/−^ subjects (1.1 ± 0.2 (*n* = 12) vs. 0.6 ± 0.1 bpm mmHg^−1^ (*n* = 8) compared to aged wild-type mice; Figure 4C, *p* = 0.02, Mann–Whitney *U* rank test). However, there was no difference found (*p* = 0.84; Mann–Whitney *U* rank test) in depressor reflex gain between aged αβγ^−/−^ and aged wild-type animals (−5.3 ± 0.3 (*n* = 6) vs. −5.3 ± 0.3 bpm mmHg^−1^ (*n* = 6, Figure 4D)).

#### 3.5.2. Baroreflex Recovery

**Figure 5:**
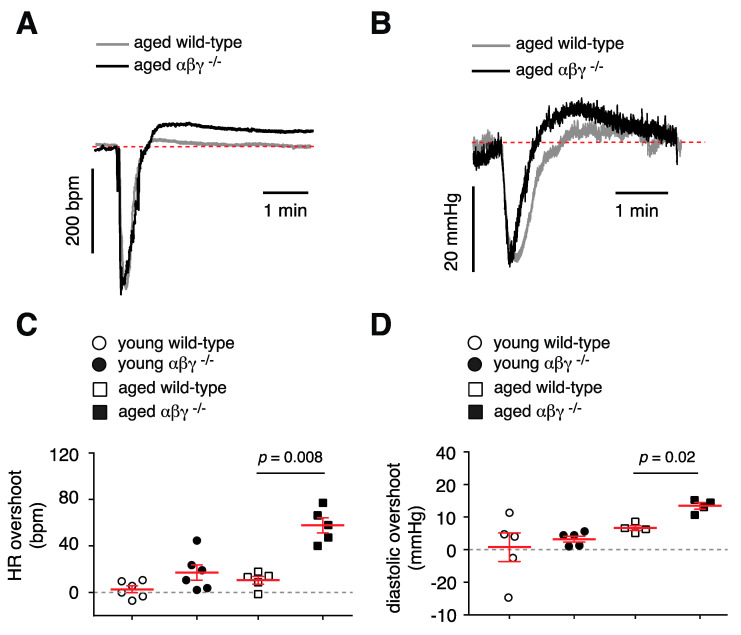
Heart rate and diastolic blood pressure restoration in αβγ^−/−^ mice. (**A**) Example trace showing heart rate after activation of the depressor reflex in aged wild-type and αβγ^−/−^ mice. (**B**) Example trace showing diastolic blood pressure after activation of the pressor reflex in aged wild-type and αβγ^−/−^ mice. (**C**) Combined data showing maximum change (overshoot) in heart rate above baseline after activation of the depressor reflex in aged, young wild-type and αβγ^−/−^ mice). (**D**) Combined data showing maximum change in diastolic blood pressure above baseline after activation of the pressor reflex in aged, young wild-type and αβγ^−/−^ mice. Means ± S.E.M were compared with a Mann–Whitney *U* rank test.

In order to gain a more complete understanding of how αβγ-synuclein deletion affects baroreflex, the recovery period after baroreflex activation was also analyzed. Following activation of the depressor reflex, restoration of heart rate was assessed in terms of a maximum change from baseline. Similarly, following activation of pressor reflex, restoration of diastolic blood pressure was assessed in terms of a maximum change from baseline. Decreases in heart rate and decreases in blood pressure following activation of the depressor and pressor reflex, respectively, were matched across genotypes and the maximum change above baseline was calculated (Figure 5A,B). The αβγ^−/−^ mice displayed a significantly higher overshoot from baseline during heart rate restoration (57 ± 7 (*n* = 5) vs. 10 ± 3 bpm (*n* = 5) in wild-type mice; Figure 5C, *p* = 0.008, Mann–Whitney *U* rank test). TKO animals also displayed a significantly higher overshoot from baseline during systolic blood pressure recovery [6 ± 1 (*n* = 4) vs. 12 ± 1 mmHg (*n* = 4) in wild-type; Figure 5D, *p* = 0.02, Mann–Whitney *U* rank test]. Again, no differences were detected between genotypes in the young animals.

## 4. Discussion

In the mammalian nervous system, the presence of other members of the synuclein proteins (i.e., β- and γ-synuclein) often makes physiological studies of functional roles of α-synuclein difficult. Therefore, synuclein triple-knockout (αβγ^−/−^) mice, by removing potential compensatory effects of β-syn and γ-syn, offers a good model to investigate the physiological function of α-syn in a synuclein-null, and an age-dependent setting [13]. Data from our study demonstrate that aged mice lacking αβγ-synuclein proteins have impaired respiratory responses to hypoxia and hypercapnia, elevated blood pressure, pressor reflex failure, and reduced ability to terminate both pressor and depressor reflex leading to overcompensation. Young αβγ^−/−^ mice showed compromised respiratory responses to hypoxic but not hypercapnic challenges. Mutations in α-syn are linked to Parkinson’s disease [4,27,28,29], and αβγ^−/−^ mice have been used as a model to study age-dependent phenotypes in Parkinson’s disease [4,13,30,31,32,33]. Moreover, recent data have linked impaired synuclein proteins to the mitochondrial dysfunction in Parkinson’s disease [34,35,36,37].

### 4.1. Respiratory Function

Recently, we have shown that during hypoxic challenge, mitochondrial Δ*Ψ_m_* in brainstem astrocytes decreases in which this depolarization of the Δ*Ψ_m_* leads to release of ROS and eventually, increases in ƒ_R_ [38]. Interestingly, in αβγ^−/−^ mice, hypoxia failed to increase ƒ_R_ from baseline levels in both young and aged mice (Figure 2), which suggests that the brainstem of these mice is hypoxic and/or the central mechanism for low-O_2_ sensing in these mice are impaired/activated [39]. Previously, we have shown that astrocytes in the ventrolateral medulla (VLM) by modulating activities of the respiratory rhythm-generating circuits of the preBötzinger complex [40,41] regulate the ventilatory response to hypoxia in an ATP-dependent manner [24,38,42]. We also showed that astroglial mitochondria can act as a central oxygen sensor [24,38]. Abnormal mitochondrial function was reported in αβγ^−/−^ mice, in which the mitochondrial membrane potential (Δ*Ψ_m_*) was found to be decreased (i.e., depolarized) in both astrocytes and neurons [43,44]. The fact that hyperoxia (33% O_2_ in the inspired air) decreased ƒ_R_ in younger αβγ^−/−^ mice but not the aged counterparts further strengthens this hypothesis.

As opposed to impaired age-independent respiratory responses to hypoxia, αβγ^−/−^ mice showed an age-dependent respiratory response to hypercapnia (6% and 9% CO_2_ in the inspired air). Our results are in agreement with age-dependent data reported from 6-hydroxydopamine (6-OHDA)-model of Parkinson’s disease [45,46,47,48] in which injection of 6-OHDA into the striatum/substantia nigra induced Parkinson’s disease in rats [45] and mice [49], and these rats also exhibited an age-dependent impaired response to hypercapnic challenges. However, 6-OHDA rats showed normal augmentation of breathing when challenged with hypoxia [45]. The fact that respiratory responses to hypoxic challenge were normal in 6-OHDA rats but were impaired in αβγ^−/−^ mice (this study) can be explained by the fact that in the 6-OHDA model, the defect is only induced in neurons [45], but both neurons and astrocytes showed impaired mitochondrial function in the αβγ^−/−^ mice [43].

### 4.2. Cardiovascular Function

Conscious measurements of blood pressure show a clear age-dependent increase in mean arterial pressure in animals lacking all three synuclein sub-types (Table 2). The baroreflex (pressor reflex) was also found to be inhibited in aged animals, as well as an increased overshoot of both heart rate and blood pressure during the recovery phase of the pressor and depressor reflexes, respectively. We did not detect any differences in heart morphometry (Table 1) between the mouse strains that could compound any differences seen in cardiovascular physiology. This was in agreement with the other report of this strain of mice being phenotypically normal compared with wild types in size, weight and gross brain anatomy, up to 14 months of age [14]. Therefore, we suggest that the data indicates the features of the cardiovascular phenotype are neurogenic in origin.

Together, the cardiovascular phenotype suggests deletion of αβγ-synuclein produces a chronic reduction of parasympathetic (vagal) tone and inability to appropriately regulate sympathetic tone as previously indicated by altered cardiac parasympathetic control [50]. The impaired parasympathetic tone can explain the exacerbation of the age-related hypertension as seen in the aged wild-type animals (compared to the young wild-type) in the present study and reported previously [51]. Parasympathetic tone has been shown to be an important determinant of blood pressure in a model of hypertension, where chronic augmentation of parasympathetic tone has been shown to be anti-hypertensive [52]. Aging of the autonomic nervous system can be characterized as a generalized shifting of the sympathetic-parasympathetic balance [53] leading to various cardiovascular pathologies, including hypertension [54]. Deletion of αβγ-synuclein produces impairment of circuits controlling the autonomic nervous system that parallels the age-related synaptic dysfunction in the striatum [13,14] and as a result, exacerbates the shift from parasympathetic to the preponderance of sympathetic tone. This is supported by the autonomic symptoms occurring before or in parallel with the movement and cognitive symptoms of the synucleinopathies [55].

This pattern of autonomic dysfunction is also supported by the impaired baroreflex displayed by aged αβγ^−/−^ mice. Baroreflex activation maintains blood pressure and heart rate within narrow physiological ranges through activation or withdrawal of central sympathetic and parasympathetic outflow in response to input from baroreceptors; decreases in blood pressure result in sympathetic activation and parasympathetic withdrawal, and blood pressure increases result in the opposite response.

The impaired pressor reflex suggests a similar dysfunction in sympathetic circuits or peripheral sympathetic signaling as sympathetic outflow must be engaged in order to restore blood pressure after it is lowered, as previously suggested [56]. Similarly, the failure of blood pressure and heart rate restoration following activation of either the pressor or depressor reflex further indicates a lack of appropriate autonomic control. This could be due to the lack of parasympathetic tone allowing sympathetic activation to dominate during baroreflex activation. This would cause an overshoot of heart rate by hyperactivation of cardiac beta-adrenoceptors when recovering from depressor reflex activation and similar inappropriate activation of vascular alpha-adrenoceptors. A similar pattern of baroreflex dysregulation was reported in mice that express a mutant form of α-synuclein [53], again suggesting that autonomic circuits are susceptible to the same pathology as seen in the striatum.

Interestingly, there was no differences in blood pressure detected in animals lacking a single synuclein or in the double (αγ) knockout animals (Table 3). This suggests that there is a significant compensation between the three members of synuclein family as all three must be absent for increased blood pressure to be observed. This is in concordance with data of synaptic dysfunction in the striatum [14].

## 5. Summary and Conclusions

The present data support a role of synuclein in the normal function of the autonomic nervous system and central respiratory control during aging. Previous data have been collected using toxin-based models of or overexpression of mutant α-synuclein proteins and have shown autonomic and respiratory deficits. However, until now, nothing has been known about the role of α-synuclein in normal autonomic physiology. We have shown αβγ^−/−^ mice have marked age-related respiratory and cardiovascular deficits. Our data suggest synuclein proteins are necessary for healthy aging in central respiratory and autonomic neuronal circuits and any treatment that reduces the availability of synuclein proteins over time may decrease the cardio-respiratory reflexes and worsen the treatment outcomes.

## Figures and Tables

**Table 1:** Comparison of morphometric parameters between αβγ^−/−^ and wild-type control animals for both young and aged groups. Means ± S.E.M were compared with Mann–Whitney *U* rank test.

	Young Wild Type	Young αβγ^−/−^	*p* Value	Aged Wild Type	Aged αβγ^−/−^	*p* Value
*n*	6	6		6	6	
Body Weight (g)	26.8 ± 0.5	28.2 ± 0.6	0.13	27.2 ± 0.7	25.6 ± 0.8	0.08
Heart Weight (g)	0.156 ± 0.005	0.157 ± 0.006	0.85	0.183 ± 0.2	0.157 ± 0.01	0.18
LV Weight (g)	0.088 ± 0.006	0.091 ± 0.002	0.79	0.113 ± 0.1	0.092 ± 0.005	0.14
HW/BW Ratio	5.78 ± 0.04	5.59 ± 0.20	0.23	6.71 ± 0.5	6.39 ± 0.4	0.53

**Table 2:** Hemodynamic parameters measured using tail-cuff plethysmograph in conscious αβγ^−/−^ and wild-type control animals for both young and aged groups. Means ± S.E.M were compared with Mann–Whitney *U* rank test.

	Young Wild Type	Young αβγ^−/−^	*p* Value	Aged Wild Type	Aged αβγ^−/−^	*p* Value
*n*	8	9		8	8	
Diastolic BP (mmHg)	86.7 ± 4.2	87.8 ± 3.4	0.94	121 ± 5.9	147 ± 6.9	0.02
Systolic BP (mmHg)	121.9 ± 5.1	125.0 ± 3.6	0.72	161 ± 7.0	185 ± 7.0	0.02
Mean BP (mmHg)	98.1 ± 4.4	99.8 ± 3.4	0.87	133.9 ± 6.2	159.5 ± 6.9	0.02
Heart rate (bpm)	568 ± 33	572.0 ±1 9	0.73	599 ± 37	632 ± 9	0.92

**Table 3:** Hemodynamic parameters measured using tail-cuff plethysmograph in conscious aged single synuclein-null (α^−/−^, β^−/−^, γ^−/−^) and the double synuclein-null (αγ^−/−^) animals. Means ± S.E.M were compared with Mann–Whitney *U* rank test.

	Aged Wild Type	Aged α^−/−^	*p* Value	Aged β^−/−^	*p* Value	Aged γ^−/−^	*p* Value	Aged αγ^−/−^	*p* Value
*n*	8	3		5		3		3	
Diastolic BP (mmHg)	119 ± 7	130 ± 6	0.38	137 ± 6	0.093	118 ± 8	0.667	120 ± 5	0.67
Systolic BP (mmHg)	159 ± 6	163 ± 5	0.92	171 ± 6	0.617	152 ± 7	0.170	155 ± 6	0.17
Mean BP (mmHg)	132 ± 6	141 ± 5	0.63	148 ± 6	0.127	129 ± 8	0.667	131 ± 5	0.55
Heart rate (bpm)	572 ± 45	636 ± 38	>0.99	651 ± 11	0.343	625 ± 49	0.667	601 ± 19	0.33

**Table 4:** Hemodynamic parameters measured under urethane anesthesia (1.2 g kg^−1^, i.v.) in αβγ^−/−^ and wild-type control animals for both young and aged groups. Means ± S.E.M were compared with Mann–Whitney *U* rank test.

	Young Wild Type	Young αβγ^−/−^	*p* Value	Aged Wild Type	Aged αβγ^−/−^	*p* Value
*n*	5	5		8	8	
Diastolic BP (mmHg)	78 ± 4	80 ± 5	0.67	72 ± 3	79 ± 4	0.71
Systolic BP (mmHg)	107 ± 3	109 ± 5	0.74	96 ± 4.0	94 ± 5	0.77
Mean BP (mmHg)	65 ± 5	67 ± 5	0.65	83 ± 4	85 ± 4	0.92
Heart rate (bpm)	658 ± 7	623 ± 22	0.22	575 ± 33	626 ± 25	0.36
